# Effect of the Physical and Chemical Characteristics of Polycarboxylate Ether Superplasticizers on the Spreading of Calcined Clays with Different Metakaolinite Contents Suspended in Synthetic Cement Pore Solution

**DOI:** 10.3390/ma19081516

**Published:** 2026-04-10

**Authors:** Suylan Matias Cruz, Ítalo Ribeiro Gonçalves Lima, Maria José Souza Serafim, Jorge Iván Tobón, João Henrique Silva Rêgo

**Affiliations:** 1 Postgraduate Program in Structures and Civil Construction, Faculty of Technology, University of Brasília, Brasília 70910-900, Federal District, Brazil; italo.lima@supermix.com.br (Í.R.G.L.); mariajss19@yahoo.com.br (M.J.S.S.); jhenriquerego@unb.br (J.H.S.R.); 2 Cement and Construction Materials Research Group, Materials and Minerals Department, Faculty of Mines, Universidad Nacional de Colombia, Medellin 050034, Antioquia, Colombia; jitobon@unal.edu.co

**Keywords:** polycarboxylate ether, superplasticizer, calcined clay, metakaolinite, synthetic cement pore solution, slump retention

## Abstract

This study investigates the influence of the physical and chemical characteristics of three polycarboxylate ether (PCE) superplasticizers—differing in main-chain length, side-chain density, and dispersing-to-stabilizing polymer ratio (75:25, 50:50, and 25:75)—on the dispersion of calcined clays with varying metakaolinite contents (30.04–74.91 wt%) in synthetic cement pore solution (SCPS). Clays were characterized by XRF, XRD, TGA, FTIR, BET, Blaine fineness, and particle size distribution; PCEs were characterized by FTIR, ^1^H NMR, GPC, and zeta potential. Dispersion was assessed via mini-slump tests for water demand, PCE dosage to achieve 260 ± 5 mm spread, and slump retention over 120 min, quantified by a normalized spread retention index (*SR*_120_). Results revealed that clays with a higher metakaolinite content (58.45–74.91 wt%) and Blaine fineness (up to 13.116 m^2^/g) required two times higher PCE dosages and exhibited greater water demand due to enhanced surface reactivity and Ca^2+^/carboxylate affinity. Slump retention depended on PCE–clay compatibility: at a low metakaolinite content (30.04 wt%), all PCEs yielded *SR*_120_ ≈ 1; at higher contents, dispersing-rich PCEs (e.g., 75:25 ratio) sustained superior retention (*SR*_120_ > 1 in intermediate cases), while stabilizing-rich variants showed rapid loss. Zeta potential values remained close to zero due to the high ionic strength of the SCPS, indicating that electrostatic interactions play only a secondary role in the dispersion process, while steric effects govern the performance of the investigated PCEs. Overall, optimal PCE selection requires matching polymer architecture to clay reactivity for effective dispersion and fluidity retention in sustainable calcined clay systems.

## 1. Introduction

Polycarboxylate ether/ester (PCE) polymers are widely recognized as one of the most significant advances in concrete technology in recent decades. They stand out for promoting high water reduction, noticeably improving workability, and achieving these effects with lower dosages compared to previous generations of superplasticizers [1]. Polycarboxylate ether-based admixtures have a “comb”-type molecule composed of a main linear chain with carboxylate and ether groups on the sides. The carboxylate groups play an essential role in the adsorption of these admixtures to cement particles [2,3]. Dispersion results from electrostatic repulsion (as observed in melamine and naphthalene-based admixtures) caused by the carboxylate groups, but is mainly influenced by the steric repulsion of the long ether side chains, which depends on the side-chain length, branching density, and concentration of PCE molecules in the solution [4,5]. The degree and duration of fluidity provided by this admixture to concrete are directly related to its structure; therefore, the shorter the main chain and the longer and more numerous the side chains, the greater and more lasting the induced fluidity will be [6]. Furthermore, the molecular weight of these additives has a significant impact on their performance. Adsorption and system fluidity increase proportionally with the molecular weight of the polymer [7].

PCE superplasticizers have demonstrated superior dispersion capabilities compared to other types of additives, such as polycondensates. Due to the diversity of adjustable structural parameters of PCE molecules, these polymers can be easily adapted for a variety of applications, providing prolonged fluidity retention, high flow velocity, and effectiveness at extremely low water/cement ratios (*w*/c < 0.25). As a result, a wide range of PCE products with distinct chemical characteristics are currently available on the market [8].

PCEs improve the workability of concrete primarily through electrostatic repulsion and steric hindrance, which disrupt the flocculation structure of the cement paste, releasing bound water into free water. In addition to interacting directly with cement, PCEs can be adsorbed onto supplementary cementitious materials, such as calcined clay, taking advantage of their positive surface charges. Even untreated natural clays can absorb PCEs when immersed in cement pore solutions [9].

Clays are made up of clay and non-clay minerals. Clay minerals are formed by the alteration of silicates present in rocks, characterized by their structures composed of tet-rahedral and octahedral sheets; therefore, they belong to the phyllosilicate subclass. Each tetrahedron contains a central atom coordinated with four oxygen atoms, and the tetrahedral rings are interconnected by shared edges, forming a two-dimensional hexagonal network. The octahedra are positioned on triangular faces and connected by shared edges to create a lattice with hexagonal symmetry, known as an octahedral sheet [1].

The pozzolanic activity of clays is generally achieved through thermal activation, with the highest reactivity usually obtained by treating the materials at temperatures between 500 and 800 °C, which are significantly lower than those required for glass formation [10].

Treatment at low temperatures causes the removal of water from the intercalated layers, leading to dehydration. Subsequently, gradual dehydroxylation occurs, with the elimination of hydroxyl groups, which causes the layers to collapse and results in an amorphous structure. The highest pozzolanic reactivity is achieved after this thermal phase when aluminum changes from coordination VI to coordination IV or V. At this time, the metakaolin contains the maximum amount of reactive silica and alumina [11], while increasing the temperature can induce structural reorganization, favoring the formation of new crystalline phases or the creation of glass.

In recent years, few studies have been conducted to understand the rheological behavior of blended cements with calcined clay and how it is altered by the addition of superplasticizers, such as PCEs [1], despite several studies having investigated the impact of calcined clays on the hydration, microstructure, and durability of cementitious materials [12,13,14,15,16,17,18].

Rheology plays a fundamental role in the application and performance of concrete. To achieve a thorough understanding, it is essential to conduct a detailed analysis involving several parameters, such as the molecular structure of the superplasticizer, the type and quantity of calcined clays, the nature and level of impurities, the specific surface area and particle size distribution of the mixture components, the surface charge of the solids, and the chemical composition of the pore solution [1].

Lei and Plank [19] compared the effect of different clay minerals in cement pastes mixed with different types of polycarboxylate (PCE) superplasticizers with the aim of obtaining an explanation for the different behaviors of clays with PCEs. Kaolinite showed a d spacing of 0.72 nm after exposure to water, and there was no change in this value after the addition of the different superplasticizers. Similarly, pure muscovite showed a d spacing of 0.99 nm, and there was no change in its spacing in the presence of superplasticizers. These results suggest that both kaolinite and muscovite interact with PCE polymers only through surface adsorption, which is aligned with the dispersion performance data, which indicate that these materials slightly affect the dispersion power of PCE superplasticizers.

Li et al. [20] evaluated the impact of a calcined clay rich in metakaolinite, present in composite cements with varying replacement rates for clinker, on the dispersion strength of different PCE superplasticizers. It was observed that the addition of calcined clay with a high metakaolinite content to ordinary Portland cement (OPC) clinker considerably hinders the achievement of adequate fluid retention. In cements of this type, conventional HPEG PCEs, based on hydrolyzable esters, along with sodium gluconate, show limited effectiveness in slump retention. This effect can be attributed to the high absorption/adsorption capacity of PCEs in the calcined clay.

Li et al. [21] investigated the PCE structures that work best in a composite cement with a high metakaolinite content. The interaction of different PCE polymers with composite cements was evaluated through zeta potential and adsorption measurements. It was found that, initially, calcined clay has a highly negative surface charge which, through the absorption of large amounts of Ca^2+^ ions from the pore solution, becomes almost neutral, which facilitates the adsorption of PCE superplasticizers. The methyl polymer (HPEG) resulted in a superior dispersion performance to methacrylate ester (MPEG) superplasticizers, both in pure calcined clay and in composite cements with calcined clay.

Although polycarboxylate ether (PCE) superplasticizers are widely used to control workability in modern cementitious materials, their performance in systems containing calcined clays remains difficult to predict. Previous studies have reported that calcined clays with high metakaolinite contents significantly increase water demand and promote rapid fluidity loss due to their high surface reactivity; however, most available investigations evaluate PCEs as chemically equivalent products or focus on composite cement systems, in which hydration reactions obscure the initial interactions governing dispersion and slump retention. As a result, practical guidelines relating the molecular architecture of PCEs—such as main-chain length, side-chain configuration, and the balance between dispersing and stabilizing polymers—to the physical and mineralogical characteristics of calcined clays are still lacking. Furthermore, in highly alkaline and ion-rich environments representative of cement pore solutions, the relative importance of electrostatic versus steric mechanisms remains uncertain, limiting the reliability of electrokinetic parameters as design tools. In this context, the present study aims to establish structure–performance relationships between PCE-based superplasticizers of the HPEG type and calcined clays with different metakaolinite contents, systematically evaluating water demand, dispersion efficiency, and slump retention in synthetic cement pore solutions.

## 2. Materials and Methods

### 2.1. Materials

#### 2.1.1. PCE Superplasticizers

The polycarboxylate additives used in this work are synthetic polymers with a molecular structure featuring a main organic chain made of polyoxyisoprene (ethylene glycol) macromonomers and grafted branches (or side chains) composed of polyethylene oxide [22]. The branches are ester-linked to acrylic units. This conformation has led to polycarboxylate molecules often being referred to as having a “comb” structure. The differences between the two polymers are the main-chain size, side-branch size and density, and molecular weight.

The additives were developed specifically for this study and are referred to in the study as MR7525, MR5050, and MR2575. This identification was used to differentiate the amount of dispersion polymer, followed by the amount of polymer needed to maintain stability:

- MR7525 (75% dispersion polymer + 25% polymer to maintain stability);
- MR5050 (50% dispersion polymer + 50% polymer to maintain stability);
- MR2575 (25% dispersion polymer + 75% polymer to maintain stability).

#### 2.1.2. Raw Clays and Calcined Clays

For the development of this work, three raw clay samples which are regularly used as supplementary cementitious materials (SCMs) were collected from cement factories located in the states of Distrito Federal, Goiás, and Ceará (Brazil). The samples, named C1, C2, and C3, were dried at 140 °C for 3 h; then, they were ground in a pan mill until all the material passed through a 200-mesh sieve (0.074 mm), homogenized in plastic bags through continuous and repetitive movements for 3 min, and separated by spatula, as proposed by Lacerda [23], for the characterization tests.

The ideal calcination temperature for the clay was 750 °C for two hours in a chamber-type kiln, determined by the mass loss of the clay minerals identified through thermal analysis (TGA/DTG/DTA).

After calcination, the calcined clay samples were crushed, homogenized, and separated, as proposed above (the same for raw clays), and named CC1, CC2, and CC3.

#### 2.1.3. Synthetic Cement Pore Solution (SCPS)

Synthetic cement pore solution (SCPS) was produced based on the characteristics of ion concentrations found in the pore solution of ordinary Portland cement pastes at early ages (<2 h) [24]. The SCPS was used to mimic the ionic environment present in cement [20,25,26].

The synthetic cement pore solution had a pH of 13.0 and was prepared by dissolving 1.72 g of CaSO_4_·2H_2_O, 7.119 g of KOH, 4.76 g of K_2_SO_4_, and 6.956 g of Na_2_SO_4_ in 1 L of MilliQ water. The ionic composition (mmol/L) of this synthetic cement pore solution (Ca^2+^ = 10; Na^+^ = 100; K^+^ = 180; OH^−^ = 127; SO_4_^2−^ = 86) is typical for normal Portland cement dispersed in water at a water/cement (*w*/c) ratio of ~0.4 [20,24,26,27].

### 2.2. Characterization of PCE Samples

The characterization of the additives was performed according to the procedures established by ABNT NBR 11768-3:2019—“Chemical additives for Portland cement concrete Part 3: Characterization tests” [28]—which defines standardized methods for evaluating the uniformity of additives regarding physical and chemical parameters. The tests included the determination of specific mass, pH, and solids content, conducted under a controlled temperature of 25 ± 1 °C.

Specific gravity was determined based on the ratio between the mass and the volume occupied by the material, using a Hubbard pycnometer for liquid additives. The values found were used to adjust the amount of water in the mixtures.

The pH measurement was performed using a digital pH meter calibrated with pH buffer solutions of 4.00, 7.00, and 9.00, according to the procedures of NBR 11768-3:2019. The liquid samples were homogenized and analyzed at a temperature of 25 ± 1 °C, with direct immersion of the electrode in the sample. The recorded value corresponded to the stabilized reading after 30 s of immersion.

The solids content was determined by oven-drying at 105 ± 2 °C to constant mass.

For the zeta potential, was used the Zetasizer Nano (Malvern Panalytical, Worcestershire, UK), taking into account 120 s to reach a temperature of 25 °C. The program uses the Smoluchowski equation for calculations. This equation relates the zeta potential to the electrophoretic mobility (velocity) of the particles. The program performs a minimum of 10 measurements and a maximum of 100. When the difference between consecutive measurements is insignificant, the program terminates the measurements. Auto Mode was used for data processing. The aqueous solution was prepared with 1 mL of superplasticizer in 25 mL of deionized water or SCPS.

FTIR measurements were performed using a Bruker Vertex 70 Fourier transform infrared spectrometer (Bruker, Ettlingen, Germany). The analysis was performed using the attenuated total reflection (ATR-FTIR) module. An average of 96 scans were performed, with a resolution of 4 cm^−1^, in the range of 400 to 4000 cm^−1^, with a background performed before measuring each sample.

The ^1^H RMN test was performed on liquid samples. The equipment used was a Bruker Magneto Ascend 600 MHZ (Bruker, Ettlingen, Germany) with a probe for liquid materials. The test was performed in a magnetic field of approximately 14T, and the equipment featured a 4.0 mm CP MAS H/X probe (BMPTech, Rio de Janeiro, Brasil). A frequency of 10 kHz was used, with a 4.25 μs pulse duration, a 10 s pulse interval, and a minimum of 1024 points to obtain each spectrum. Tetramethylsilane (TMS) was used as the internal standard, and the solvent used was deuterated chloroform (CDCl_3_) with δ = 7.29 ppm. The data obtained were analyzed using Topspin 4.5.0 software. This software allows spectra to be deconvoluted using a Gaussian/Lorentzian function.

The recommended sample size is 5 to 10 mg of material.

In the analysis of organic molecular structures, NMR is a powerful and reliable technique, providing detailed information on molecular architecture. This method enables the identification of hydrogen- and carbon-containing environments and offers insights into functional groups, structural units, and the connectivity between different segments of a macromolecule [29]. Magnetic resonance spectroscopy was applied to ^1^H nuclei for the structural characterization of the polymers of the superplasticizer additives.

Gel Permeation Chromatography (GPC) was used to determine the molecular weights (M_w_ and M_n_), Polydispersibility Index (PDI), and conversion rate of polymer samples in a separation module equipped with three columns in series (KF-802.5, KF-804L, and KF-805L, Resonac Corporation, Tokyo, Japan), compatible with tetrahydrofuran (THF) solvent, at a concentration of 1 mg/1 mL. The equipment used was the Malvern Instruments Viscotek RImax (Malvern Panalytical, Worcestershire, UK).

### 2.3. Characterization of Raw Clay and Calcined Clay Samples

The chemical characterization was performed using the XRF method, obtaining results for Al_2_O_3_, CaO, Fe_2_O_3_, K_2_O, MgO, Na_2_O, SiO_2_, and TiO_2_. The calculation of the alkali content is expressed in alkaline equivalent in Na_2_O (Na_2_O_eq_), according to Equation (1) [30].

(1) $${Na}_{2}O_{eq}={\%Na}_{2}O+0.658*{\%K}_{2}O$$

Pressed pellets were prepared using 1.000 g of sample and 5.000 g of Cera Max as a binder. This mixture was homogenized and pressed with a 20 kton press for 11 s. The pellets were analyzed using a wavelength-dispersive X-ray fluorescence spectrometer, a Malvern Panalytical 4 kW Zetium (Malvern Panalytical, Worcestershire, UK). The Super Q6 software was used in a semi-quantitative Omnian application, which allows exploratory analysis for unknown chemical compositions. The values presented were normalized to 100%, incorporating the previously measured loss on ignition.

The mineralogical characterization of the clays was performed by XRD of the total sample. The samples were analyzed in a RIGAKU equipment, model ULTIMA IV (Rigaku Corporation, Tokyo, Japan), which operates with a copper tube and nickel filter, under a voltage of 35 kV and a current of 15 mA, a scanning speed of 2°/minute, and steps of 0.05°. The analyses were performed in the 2θ range from 2° to 60°. Mineral identification was performed using the JADE 9.0 program, Windows-based, with a PC-PDF database (Powder Diffraction File—PDF for PC/ICDD).

FTIR measurements were performed as described in Section 2.2.

The clay samples were subjected to thermoanalytical tests (DTA/DTG/TGA), which were used to quantify the dehydroxylation of kaolinite, following well-established procedures for cementitious materials [31], using TA Instruments SDT 650 equipment (TA Instruments, New Castle, DE, USA), ranging from 40 °C to 1000 °C, with a heating rate of 10 °C·min^−1^ under a nitrogen atmosphere (N_2_). The calculation of the kaolinite content (wt%) of each clay sample was performed using Equation (2), thermogravimetric analysis (TGA), and the mass loss in the kaolinite dehydroxylation interval between 400 and 600 °C [32,33,34].

(2) $${wt.\%}_{kaolinite}={wt.\%}_{kaolinite-OH}*\frac{M_{kaolinite}}{2M_{water}},$$

defined as ${wt.\%}_{kaolinite-OH}$, where $M_{kaolinite}$ and $M_{water}$ represent the molar mass of kaolinite and water, respectively.

The metakaolinite calculation was made using the difference between the kaolinite content of the raw sample and the sample after the calcination process. Thermogravimetry allowed the analysis of the dehydration and dehydroxylation temperatures of natural clays, helping to determine the calcination temperature and analyze the efficiency of calcination in the dehydroxylation of calcined clay, in addition to helping to determine the kaolinite and metakaolinite contents of the samples.

The density test was performed using Pentapyc 5200c equipment (Quantachrome Instruments, Boynton Beach, FL, USA). The calcined clay samples were oven-dried at 100 °C for 24 h before the test, since moisture adsorbed to the powder impairs the equipment’s reading. The determination of the specific mass of the solids was performed in accordance with ABNT NBR 17212:2025 [35], which establishes the method for calculating the specific mass (or true density) of the fraction of soil passing through a sieve with a 2.0 mm opening. The test is based on measuring the mass of a dry sample and the volume it occupies when immersed in distilled water using a pycnometer.

The liquidity limit (LL), which corresponds to the soil moisture content at the boundary between the liquid and plastic states, was determined according to NBR 6459 (2025) [36].

The plasticity limit (LP), which corresponds to the soil moisture content at the boundary between the plastic and semi-solid states, was determined according to NBR 7180 (2025) [37]. The plasticity index (PI) was calculated by the difference between the liquidity limit and the plasticity limit, providing an indication of the soil’s plasticity.

The BET specific surface area allows determination of the specific surface area of very fine materials with particles smaller than those of cement. It was used to evaluate calcined clays, complementing their physical characterization. The test was conducted following the procedures set out in ISO 9277 (2022) [38], using the Nova Station equipment (Anton Paar, Graz, Austria).

Fineness determination was performed in accordance with ABNT NBR 16372:2015—Portland cement and other powdered materials—Determination of fineness by the air permeability method (Blaine method) [39].

The particle size distribution tests were performed using a Malvern Mastersizer 3000 E granulometer (Malvern, Malvern, UK) to verify particle size distribution in the range of 0.04 μm to 2.5 mm, using ethyl alcohol as a dispersant.

### 2.4. Dispersion Analysis of Calcined Clays in SCPS

The rheological properties of all calcined clay samples dispersed in SCPS were determined using the mini-slump test [40,41,42]. To prepare the samples, 100 g of calcined clay was placed in a metal container containing SCPS for 1 min, then mechanically agitated for 1 min, left to rest for 1 min, and agitated again for another minute [26]. The mixer used was a Hamilton Beach soil spreader (Hamilton Beach Brands Holding Company, Glen Allen, VA, USA), with a speed of 10,000 rpm, composed of 19 mm propellers and a metal cup 178 mm high, with a base and top opening of 65 mm and 95 mm in diameter, respectively, and which had fixed baffles. Immediately after agitation, the slurry was deposited into a truncated cone with an upper diameter of 19 mm, a lower diameter of 38 mm, and a height of 57 mm. This slurry was placed on a slumping table, filled to the brim, and the cone was removed. The resulting diameter represented the slurry flow value. The average value of two perpendicular diameters measured with a caliper was recorded as the test result [43]. The SCPS/clay ratios required to obtain a slump flow were adjusted to the value of 180 ± 5 mm [24,26].

In the samples to be added, the PCE superplasticizers were pre-dissolved in the SCPS, and the amount of water contained in the PCE solution was subtracted from the amount of SCPS used in the mixture. The PCE dosages were varied to achieve a flow rate of 260 ± 5 mm or higher [24]. Finally, the slump retention was measured every 30 min, until 120 min had elapsed.

To synthesize the different slump loss behaviors observed into a single quantitative descriptor, the normalized spread retention index (*SR*_120_) was defined using Equation (3).

(3) $${SR}_{120}=\frac{D_{120}-D_{Ref}}{D_{0}-D_{Ref}}$$

where

*D*_0_ = initial spread (≈260–265 mm);

*D*_120_ = spread after 120 min;

*D_ref_* = minimum reference spread (180 mm, total loss of fluidity condition).

The zeta potential of calcined clays in SCPS with and without PCE superplasticizers was determined in the same conditions as those used for the superplasticizer samples. The aqueous solution was prepared with 1 g of solids in 25 mL of SCPS or 1 g of solids + 1 mL of superplasticizer in 25 mL of SCPS.

## 3. Results and Discussion

### 3.1. Characterization of PCE Samples

The results for the specific mass, pH, and solids content of the additives are presented in Table 1.

From the results, it was possible to observe that the three additive samples presented similar density (1.086–1.093 g/cm^3^), solids content (48.14–48.61%), and pH (4.07–4.73) values. Ribeiro et al. [44] evaluated the effects of polycarboxylate-based superplasticizers on the hydration, rheology, mechanical strength, and environmental evaluation of LC^3^ (Limestone Calcined Clay Cement); the additives used for them presented specific mass and pH values of 1.09 g/cm^3^ and 5.5, respectively, and the solids content of the two additives was 43.5 and 47.0 wt%, values close to those presented by the additives used in this work.

**Figure 1:**
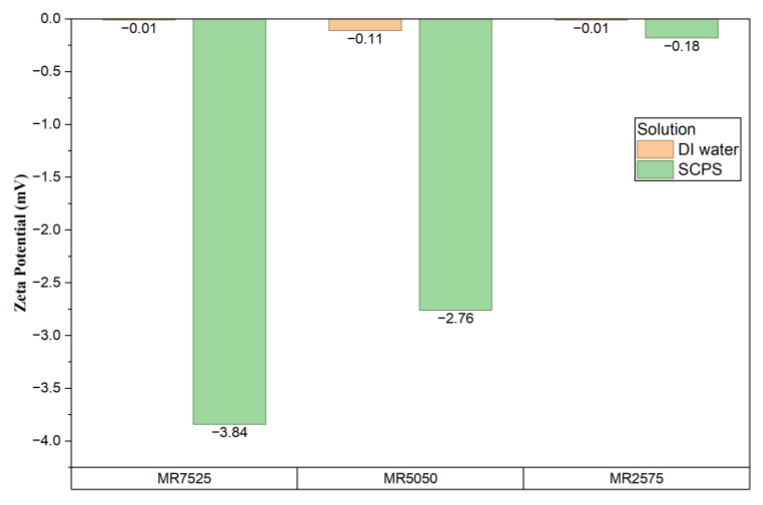
Zeta potentials of PCE superplasticizers.

The zeta potentials of superplasticizers in deionized water and SCPS are presented in Figure 1.

The zeta potential results show that the additives exhibit values close to zero in deionized water but become slightly more negative in synthetic cement pore solution (SCPS), especially MR7525 and MR5050. This increase is consistent with studies demonstrating that the high alkalinity and high ionic strength of a pore solution promote greater deprotonation of carboxylate groups and conformational alteration of anionic superplasticizers, increasing their effective charge in solution [45]. The compression of the electrical double layer in highly conductive media, described by Menon et al. [46], also contributes to variations in zeta potential and to limitations in the quantitative interpretation of absolute values.

The differences between the additives reflect their distinct molecular architectures: MR7525 and MR5050 respond more intensely to SCPS conditions, suggesting a higher density of ionizable groups or less steric shielding, while MR2575 shows much less variation. This behavior aligns with structural and electrokinetic analyses described by Hirata et al. [47], which relate functional density and side-chain configuration to electrophoretic behavior and interaction with cementitious phases. Thus, the zeta potential constitutes a useful, albeit complementary, indicator for understanding performance differences between additives in high-alkalinity systems.

**Figure 2:**
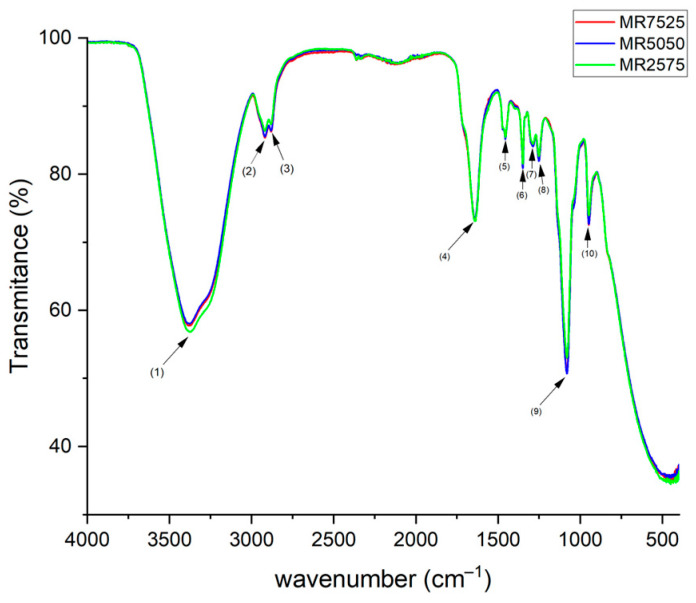
FTIR of PCE superplasticizers.

The results of infrared spectroscopy (FTIR) for the PCE superplasticizers are presented in Figure 2. Table 2 shows the identified bands and their respective functional groups.

The absorption peaks around 3377 cm^−1^ correspond to O–H and N–H stretching vibrations of bonds in the HPEG [48]. The absorption peaks at 2920 and 2883 cm^−1^ can be attributed to the stretching vibration of the C–H bonds of aliphatic groups [20,43,44,49,50]. The absorption peak at 1640 cm^−1^ was generated by stretching vibration of carboxylate C=O [44]; however, Li et al. [20] state that this peak is attributable to the adsorption vibration band of –C=C– in the HPEG molecule. The peak at 1460 cm^−1^ corresponds to the bending vibration peak of –CH_2_ [43,49]. The peak at 1349 cm^−1^ is attributed to the stretching vibration of –CH_3_. The peak relative to the CH stretching vibration band is found at 1288 cm^−1^ [44]. The peak at 1251 cm^−1^ corresponds to the C–O stretching band [49]. The characteristic absorption peak of the C-O-C ether bond in the ethylene oxide side chain appears at 1081 cm^−1^ in HPEG [20,48,50,51], and the peak at 949 cm^−1^ corresponds to the –COO^−^ groups [44].

**Figure 3:**
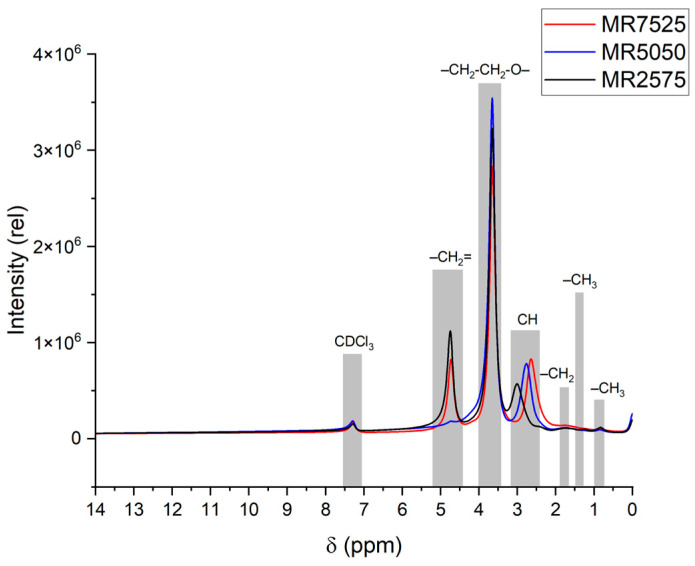
^1^H NMR spectra of PCE superplasticizers.

Figure 3 and Table 3 show the ^1^H NMR spectra of the HPEG superplasticizers and the chemical shifts and integral areas, respectively.

The signals in the δ = 4.67–4.75 ppm region correspond to vinyl protons (–CH_2_=) in the final unsaturated molecular chain of HPEG [29,52]. MR5050 showed the smallest area (3523), indicating a lower amount of residual unsaturation. MR7525 showed the largest area (5999), consistent with its higher dispersion polymer content. This behavior suggests that additives with a higher fraction of dispersion polymers tend to exhibit greater surface reactivity, favoring initial adsorption on cementitious particles.

The largest area of the signals at δ = 3.65–3.66 ppm, which correspond to the repeats of the monomer (–CH_2_-CH_2_-O-) [29,51,53,54,55,56,57,58,59,60,61,62,63], was predominant in all superplasticizers, confirming the large number of repeats of polyethylene glycol units in the side chain [29,59]. It was observed that the area of this signal is practically constant among the additives (~14,500), suggesting that the number of side chains is similar between dispersion and maintenance polymers.

The signals in the δ = 2.63–3.00 ppm range, associated with the protons of the (CH) groups of acrylic acid [55], varied according to the composition. MR2575 showed the highest relative intensity (8246), indicating a higher proportion of (CH) groups, possibly linked to the ester group of the bond between acrylic acid and the polyol, which makes it compatible with the formulation richer in maintenance polymers.

In turn, the signals at δ = 1.73–1.84 ppm, which correspond to the protons of the (–CH_2_) group of the main chain [60,64], also showed variation according to the composition. MR7525 showed the highest intensity area (8829), which means a higher proportion of methyl hydrogens (–CH_2_) in the main chain, possibly originating from groups close to the main chain’s bonds with carboxylic groups, which favors initial adsorption, relative to additives with a higher proportion of dispersion polymers.

The signals at δ = 1.33–1.55 ppm correspond to the protons of the (–CH_3_) group in the main chain [65], and they showed the highest area for the superplasticizer MR2575, which relates these signals to superplasticizers with a higher proportion of maintenance polymers, probably representing the (–CH_3_) groups close to the groups attached to the side chain.

Finally, the signals at δ = 0.8–0.9 ppm are associated with the (–CH_3_) groups of the PCE side chain [52,53,55,61,62,64]. The superplasticizer MR2575 has a larger area (3676), which may suggest a greater number of terminal methyl groups belonging to PEG units of the side chain.

Quantitative analysis of the integrated areas of the ^1^H NMR spectra allowed a direct comparison of the main chain and the side chains of the PCEs. The sum of the areas attributed to the main chain (4.67–4.75, 1.7–1.8, and 1.3–1.5 ppm) corresponded to approximately 43.1% for MR7525, 38.4% for MR5050, and 35.1% for MR2575, indicating a relatively longer main chain in MR7525. In contrast, the areas associated with the side chains (3.65 ppm and 0.8–0.9 ppm) represented about 40.9% in MR7525, 44.8% in MR5050, and 44.7% in MR2575, evidencing a higher density of branching in MR2575. These results quantitatively confirm that MR7525 exhibits an architecture dominated by a longer back chain, while MR2575 is the most branched polymer, with MR5050 exhibiting intermediate behavior.

The GPC results presented in Table 4 revealed marked differences in the molecular weight distributions of the three PCE superplasticizers.

MR2575 presented the highest M_w_ (19.892 g·mol^−1^) and the highest apparent conversion rate (88.37%), indicating a larger fraction of macromonomers effectively incorporated into the polymer backbone. However, this additive also exhibited the highest polydispersity index (1.343), suggesting a broader distribution of chain lengths and a more heterogeneous molecular architecture. In contrast, MR5050 showed the lowest M_w_ (14,241 g·mol^−1^) and the lowest apparent conversion (74.20%), reflecting a higher proportion of low-molecular-weight species and unreacted macromonomers, accompanied by lower PDI (12.241). MR7525 displayed a low M_w_ (12,749 g·mol^−1^) and conversion (79.00%), combined with the low PDI (1.249), indicating a more controlled polymerization process and a narrower molecular weight distribution. These results demonstrate that, beyond molecular weight, differences in conversion efficiency and dispersity play a key role in defining the intrinsic molecular architecture of the PCEs.

It can be noticed that all the polymers have low PDI values, which are characteristic of high-quality PCEs with a narrow molecular distribution [49,64].

The three HPEG-based PCE superplasticizers exhibited similar macroscopic properties, such as density (1.086–1.093 g/cm^3^), solids content (48.14–48.61%), and pH (4.07–4.73%), allowing for direct performance comparisons. However, molecular and electrokinetic analyses revealed significant differences: FTIR and ^1^H NMR identified variations in the main-chain composition, relative proportions of dispersing and stabilizing polymers, and distribution of functional groups. Quantitative integration of the ^1^H NMR spectra showed that the relative contribution of the main chain (vinylic and aliphatic backbone protons) ranged from approximately 43.1% (MR7525) to 38.4% (MR5050) and 35.1% (MR2575), while the contribution of side chains increased from about 40.9% (MR7525) to ~44.8% (MR5050) and ~44.7% (MR2575), evidencing increasing branching density. Accordingly, MR7525 exhibited a higher fraction of dispersing polymers, characterized by stronger relative contributions from backbone methylene and vinylic protons, indicating a relatively longer main chain and enhanced initial adsorption, whereas MR2575 showed a higher proportion of holding polymers, reflected by stronger terminal methyl and acrylic proton signals, associated with higher functional density and delayed dispersion effects. MR5050 exhibited an intermediate architecture. Zeta potential measurements indicated low electrokinetic magnitudes in SCPS due to double-layer compression, highlighting that the distinct dispersing behaviors are mainly governed by steric effects. Trends in GPC corroborate NMR, with MR2575 showing a higher molecular weight (19.892 g/mol), apparent conversion (88.37%), and dispersity (1.343), indicating longer and more heterogeneous chains; MR5050 a lower conversion (74.20%) and PDI (1.225), reflecting low-molecular-weight segments; and MR7525 a narrow distribution (PDI 1.249), confirming that the molecular architecture is determined not only by molecular weight but also by the degree of macromonomer incorporation and uniformity of chain growth. Thus, GPC and NMR provide complementary and coherent evidence on the structures of PCEs.

### 3.2. Characterization of Raw Clay and Calcined Clay Samples

The results of the chemical and physical characterizations of the raw clays and calcined clays are presented in Table 5.

The calcined clay samples presented silica (SiO_2_) contents of 52.00% (CC1), 67.00% (CC2), and 53.00% (CC3). Silica is a common component in clays, between 40% and 80%, and plays an important role in the formation of the structure and properties of clays [66].

The alumina (Al_2_O_3_) contents were 43.00% (CC1), 24.00% (CC2), and 39.00% (CC3). Alumina is a key component in clays, and its concentration (between 10 and 40%) can affect the physical and chemical properties of clays, such as water retention capacity and plasticity [67]. The sums of the fractions of silica (SiO_2_), alumina (Al_2_O_3_), and iron oxide (Fe_2_O_3_) are 96.30% (CC1), 95.30% (CC2), and 96.60% (CC3), values well above the minimum required by standard NBR 12653/2015 [68] for class N of pozzolanic materials, which is ≥70%. SiO_2_ contents are associated with the presence of clay minerals and quartz, while Al_2_O_3_ contents are related to the presence of the clay mineral kaolinite, muscovite, and feldspars [69]. The Al_2_O_3_/SiO_2_ ratio for the three clays was 0.83 (CC1), 0.36 (CC2), and 0.74 (CC3). These values differ significantly from the value of 0.86 for theoretical kaolinite clays, and it is not possible to confirm the presence of the clay mineral kaolinite for the clays studied from this ratio [70], or at the very least they are low-grade.

The alkali content of each clay sample was calculated according to Equation (1). The estimated alkali contents, expressed in alkaline equivalent in Na_2_O (Na_2_O_eq_), in each sample were 0.49% (CC1), 1.92% (CC2), and 0.96% (CC3). With the exception of sample CC2, these values are well below the minimum required by standard NBR 12653/2015 for class N of pozzolanic materials, which is ≤1.50%. High concentrations of Na^+^ and OH^−^ ions raise concerns regarding the use of various materials in the production of concrete and mortar, as they influence the alkali–aggregate reaction [33].

The presence of Fe_2_O_3_ in a sample is related to the possible isomorphic substitution of iron for aluminum in the octahedral layer of the clay minerals. The presence of K_2_O could be related to the presence of muscovite/illite, while Na_2_O and MgO could be associated with the presence of montmorillonite and feldspars [33]. The concentrations of these elements can influence specific characteristics of the clays, such as cation exchange capacity, color, and strength, among other properties. They can be presented both as impurities and as essential components in the composition of the clay [71].

**Figure 4:**
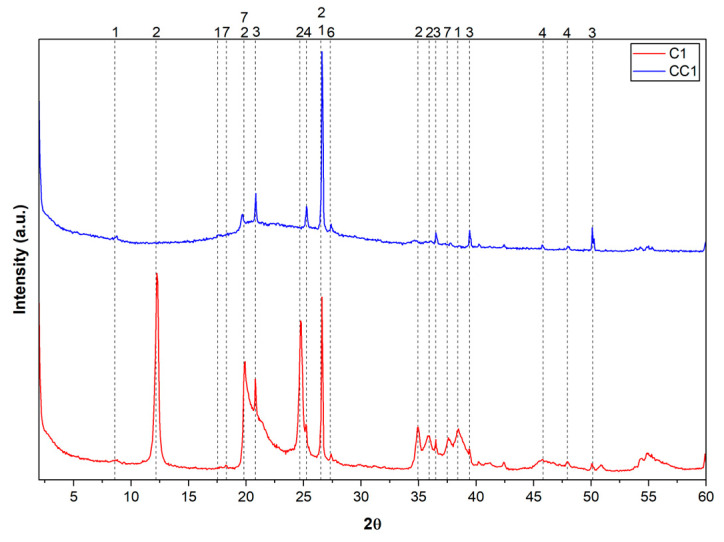
XRD of raw clay C1 and calcined clay CC1.

**Figure 5:**
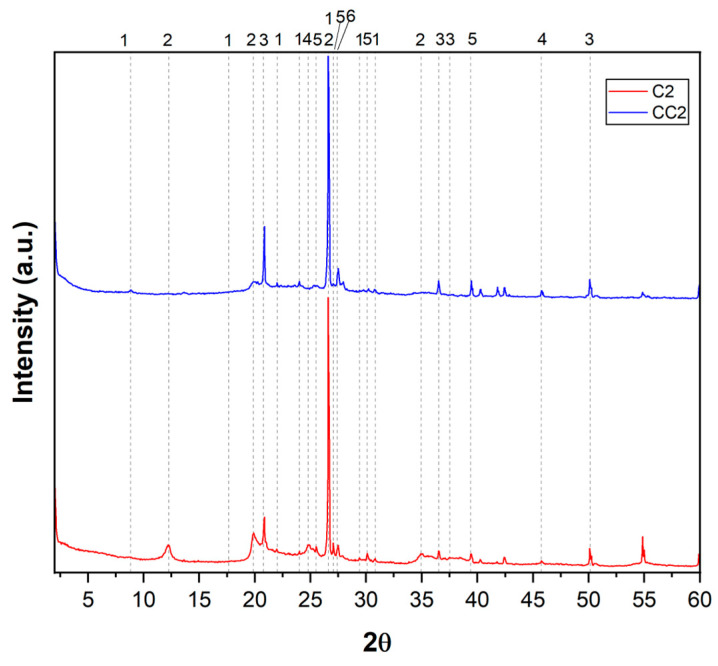
XRD of raw clay C2 and calcined clay CC2.

**Figure 6:**
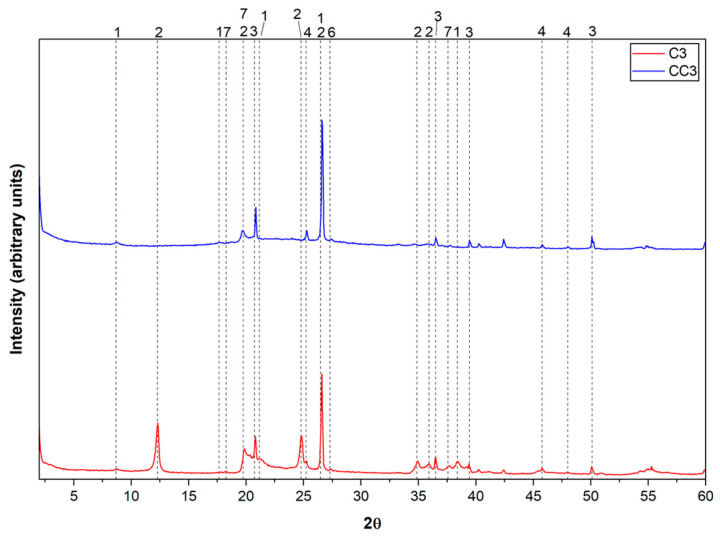
XRD of raw clay C3 and calcined clay CC3.

Chemical analysis provides important data on the composition of clays, but it is essential to also consider other aspects, such as mineralogy, for a more comprehensive understanding of their properties. To identify the minerals present, an X-ray Diffraction (XRD) test was performed on the in natura and calcined samples. The peaks of the diffractograms were identified using the database reference databases for XRD and the International Center for Diffraction Data (ICDD), and they are presented in Table 6, while the clay diffractograms are shown in Figure 4, Figure 5 and Figure 6.

Based on the data obtained in the X-ray Diffraction (XRD) test, it was possible to identify the presence of minerals such as quartz, kaolinite, and anatase in all in natura samples analyzed. It was observed that quartz presents high intensity peaks, being predominant in the samples. All natural samples presented well-defined peaks of kaolinite, in addition to signals, albeit less intense, of muscovite. The presence of muscovite is in accordance with the results obtained in the chemical analysis by XRF, since the detection of K_2_O can be related to this mineral.

Quartz, a mineral widely present in various types of clay, was identified by its characteristic peaks in the XRD, suggesting its occurrence in all samples. Composed of silicon dioxide (SiO_2_), it is known for its high hardness and chemical stability. Its presence influences important physical properties of clays, such as mechanical strength and water retention. Sample CC2, in particular, presented a more prominent quartz peak, suggesting a higher concentration of this mineral [72]. Kaolinite, a typical clay mineral with particles around 0.7 microns in diameter and 0.5 microns in thickness, has a layered structure and is widely recognized for its contribution to the plasticity of clays. The peaks observed in XRD confirm its presence in all in natura samples.

Composed mainly of silicon and aluminum, kaolinite influences characteristics such as the shrinkage, plasticity, and mechanical strength of clay materials [71]. The identification of quartz, kaolinite, and muscovite indicates a relatively common mineralogical composition among the samples, although the proportions of these minerals vary significantly, which can directly impact their properties and applications [71,72,73,74]. Variations in the height of the diffractometric peaks reinforce these differences. The kaolinite and muscovite peaks presented different intensities, indicating that these clay minerals were distributed in different proportions among the samples. Such variations may influence properties such as the calcination temperature required for the dehydroxylation of clays [15].

After calcination, it can be observed that the kaolinite peaks disappear, confirming the dehydroxylation of the clay mineral, which is associated with its thermal activation.

**Figure 7:**
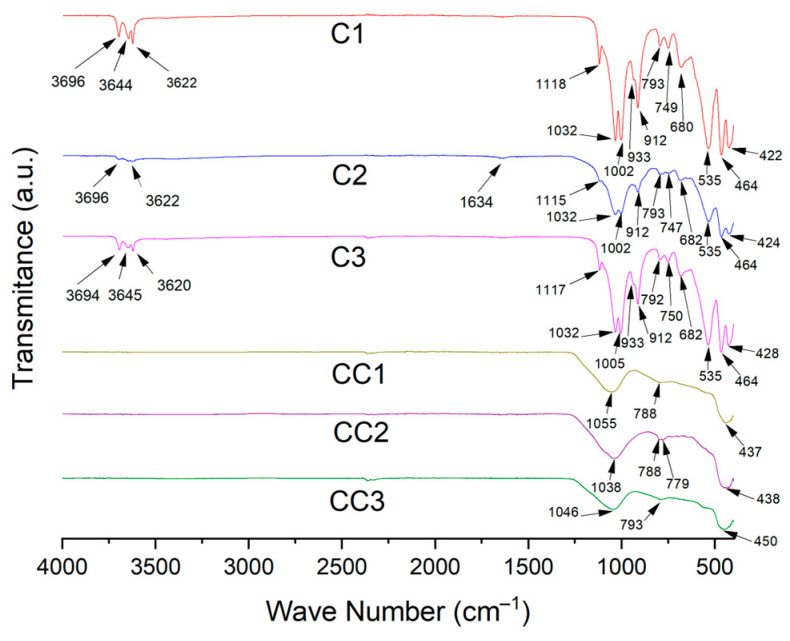
FTIR of raw clays and calcined clays.

The FTIR spectra of the clay samples are presented in Figure 7.

Typically, clay minerals present several characteristic absorption bands due to their structural composition. Samples C1 and C3 presented strong and sharp O–H and Si–O stretching peaks at 3695–3620 cm^−1^ and 1115–1010 cm^−1^, respectively, due to the ordered nature of the hydroxyl groups typical of kaolinite, but in sample C2 these peaks were weaker. The broader and less sharp O–H stretching bands, around 3620–3550 cm^−1^, due to the presence of interlayer water, indicated a more disordered structure of illite/muscovite.

The hydroxyl bending peak observed at 1638 cm^−1^, accompanied by the peak at 912 cm^−1^, reveals the presence of illite/muscovite [75,76,77,78]. The presence of smectite can be ruled out due to the absence of the peak at 3426 cm^−1^ [79]. Samples CC1, CC2, and CC3 did not present peaks in the water-related bands, confirming the heat treatment observed in the XRD analysis. Quartz interference was detected in the peaks at 1007, 695, 539, and 471 cm^−1^ [80].

**Figure 8:**
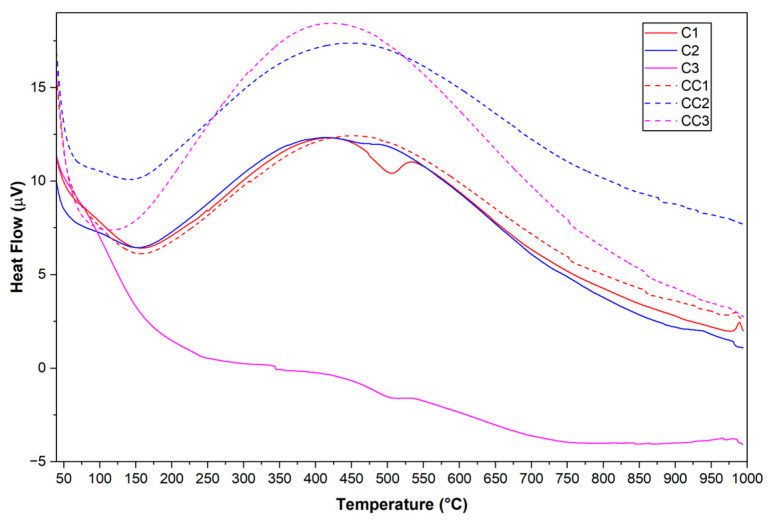
Curves of thermal analysis of samples by DTA.

**Figure 9:**
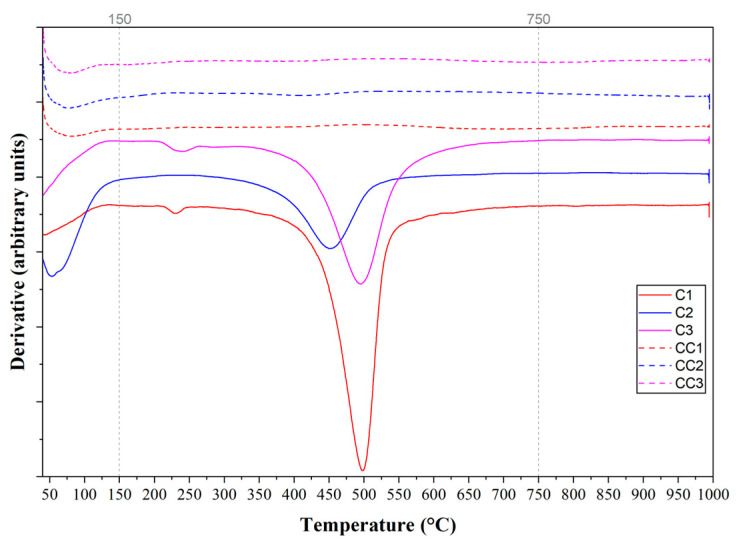
Curves of thermal analysis of samples by DTG.

**Figure 10:**
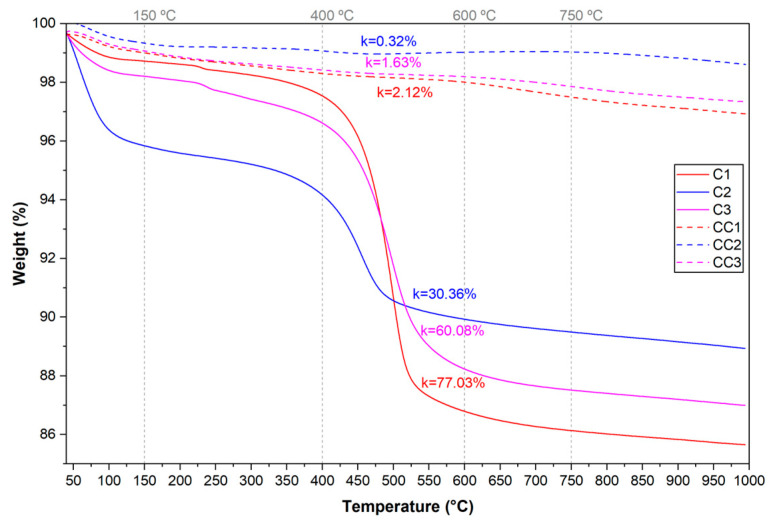
Curves of thermal analysis of samples by TGA.

The DTA curve represents the heat absorption and exothermic behavior during heating [81], while the numerical derivation of the TG curve provides a DTG graph used to determine the temperature at the maximum peak and other important peak parameters [80], and thermogravimetric analysis (TGA) measures the changes in weight loss [81,82]. Figure 8, Figure 9 and Figure 10 present the curves of the thermal analyses of the clays used in the study.

In Figure 8, it can be observed that the first endothermic peak occurs, for clay C1, below 200 °C, referring to the dehydration of the samples, due to the loss of free water [70,83,84]. Sample C3 presents the first endothermic peak between 250 and 350 °C, a range in which pre-dehydroxylation occurs, that is, water loss continues to occur in the samples [70]. In the temperature range between 450 and 550 °C, another endothermic peak can be observed in the samples, initiating the dehydroxylation reaction of kaolinite and transformation into metakaolinite. Finally, an exothermic peak can be observed above 950 °C. At this temperature, recrystallization reactions and the appearance of mullite occur [81,85,86].

In Figure 9, it can be seen that the DTG curve has the first peak below 150 °C, in the temperature range that indicates the loss of free water. A second peak can be observed at 200 and 270 °C (C1 and C3), which corresponds to the dehydroxylation range of aluminum hydroxide, present in an unidentified phase of clay mineral, probably gibbsite. Finally, it can be observed that between 400 and 550 °C is the temperature range in which the dehydroxylation of kaolinite begins. The temperature of 750 °C is the end of the event for all samples, from which it is possible to estimate the ideal calcination temperature of the material.

When observing the TGA curves in Figure 10, the dehydroxylation of the clay minerals in the samples occurs up to a temperature of approximately 750 °C, as shown by the DTG curves. The mass losses at this temperature are 12.59% (C1), 6.35% (C2), and 10.69% (C3), the mass losses being calculated in the temperature range of 150 to 750 °C. The kaolinite content of each clay sample was calculated according to Equation (2), from the mass loss during the kaolinite dehydroxylation interval, between 400 and 600 °C [32,33,34]. The estimated kaolinite contents in each sample were 77.03% (C1), 30.36% (C2), and 60.08% (C3).

The determination of kaolinite content by TGA may be influenced by possible interference from secondary phases present in the clay, such as muscovite or illite, which also undergo partial dehydroxylation processes at similar temperatures [87]. However, data obtained by XRD indicate that these phases are present in reduced quantities and that the dehydroxylation associated with them occurs in a wider thermal range [33].

The natural clays C1, C2, and C3 presented densities of 2.60, 2.50, and 2.65, respectively. After calcination, the calcined clays CC1, CC2, and CC3 showed little variation in their density, with values of 2.60, 2.57, and 2.60, respectively. These values are similar to the density of metakaolinitic clay samples reported in the scientific literature [15,24,44,88].

The results showed that the natural clays presented high plasticity indices (17–26%), with values of 17, 21, and 26% for C1, C2, and C3, respectively. After calcination, it can be observed that the calcined clays CC1 and CC3 showed a decrease in the plasticity index with values of 10 and 11%, respectively. Clay CC2, however, showed a loss of this property after calcination, indicating that this sample contains low amounts of clay minerals [89].

The BET surface area values (C1–C3: 27.8, 48.0, 31.7 m^2^·g^−1^; CC1–CC3: 48.4, 33.7, 28.4 m^2^·g^−1^) show a heterogeneous response to calcination. While CC1 exhibits a substantial increase, CC2 and CC3 show a reduction in area. These variations are consistent with previously reported results, according to which the effect of calcination on specific surface area depends critically on the starting mineralogy, the treatment temperature range, and the mechanisms of collapse or pore formation, such as metakaolinite formation [90] and the contribution of other mineral phases to the physical properties [91].

Academics tend to prefer the BET method, due to its fundamental basis, over the Blaine method, due to its semi-empirical nature. However, the cement industry relies extensively on Blaine density because of its more consistent results than nitrogen adsorption density, the determination of which, moreover, takes longer [92]. As shown in Table 4, the calcined clays CC1, CC2, and CC3 presented values of 9.720, 7.438, and 13.116 m^2^·g^−1^, respectively. Since the Blaine method measures the external surface area of the particles, the results were consistent with the PSD results presented in Figure 9, where clays with a greater fineness Blaine exhibited a smaller average particle size.

**Figure 11:**
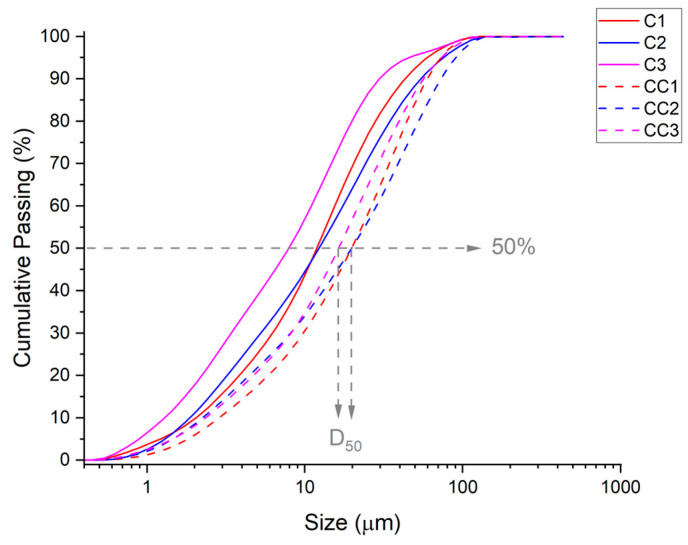
Particle size distribution (PSD) of the raw clays and calcined clays.

The average particle diameter increased with the calcination of the samples (Figure 11), confirming that heat treatment tends to agglomerate the particles [93].

The presented results are similar to those of Schmid and Plank [24], as the clay minerals yielded D_50_ values of 14.8 and 19.2 μm. The D_50_ and D_m_ values were significantly higher than those reported by Ribeiro et al. [44,49] for calcined clays from Brazil.

### 3.3. Water Demand

**Figure 12:**
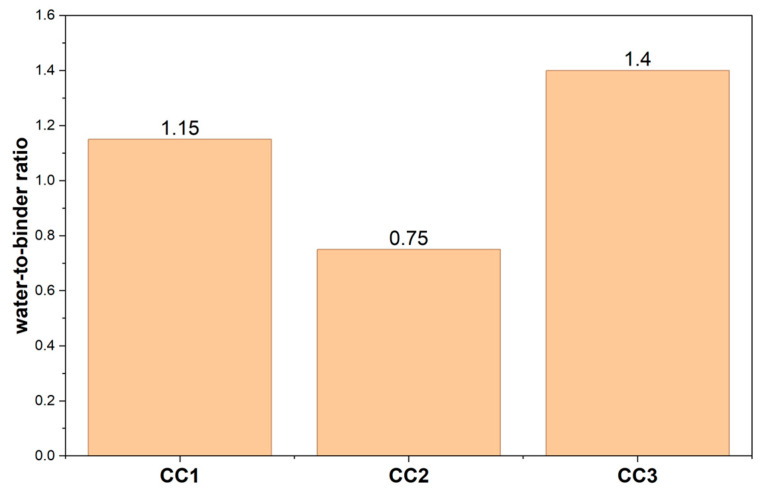
Water demand of calcined clays for spread flow 180 ± 5 mm; no PCE polymer added.

The water demand of calcined clays and its impact on the workability of calcined clay pastes, prepared in SCPS, were evaluated. Figure 12 presents the water demand to achieve a spread of 180 ± 5 mm.

It can be observed that CC2, which has a lesser Blaine fineness, showed a lower water demand (0.75) to achieve the desired fluidity, while CC3, with a greater Blaine fineness, showed a higher water demand (1.40) to achieve fluidity. This indicates that the water demand of the calcined clay samples investigated here increased, presumably resulting from the increase in Blaine fineness. These results are consistent with the range of water demands found by other authors [20,24].

On the other hand, when compared, the specific Brunauer–Emmett–Teller (BET) areas and the water demands of the clays do not show a correlation, similar to what was found by Schmid and Plank [24]. This is probably due to the specific BET area being directly related to the morphology of clay mineral particles [94].

### 3.4. Dispersing Performance and Slump Retention

**Figure 13:**
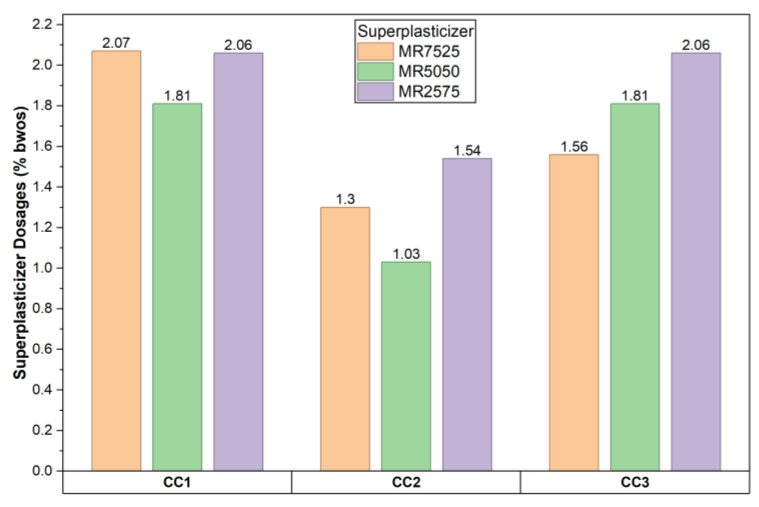
Dosages of PCE superplasticizers required to obtain a calcined clay paste spread flow of 260 ± 5 mm.

The dispersing capacity of the three superplasticizers in pure calcined clay suspensions, prepared in SCPS, was evaluated by measuring the spread of the paste as a function of dosage. The PCE samples showed quite diverse performance. The additions of the superplasticizers allowed for very high fluidity, expressed by a spread of 260 ± 5 mm, all with quite high dosages (Figure 13), especially for the CC1 and CC3 clays. These high dosages are mainly related to the high Blaine fineness and metakaolinite content of these clays; however, differences among PCEs can also be observed. In systems with higher clay reactivity, MR7525 systematically required a lower relative dosage increase compared to MR2575, which is consistent with its longer effective main chain and stronger initial steric dispersion capacity.

**Figure 14:**
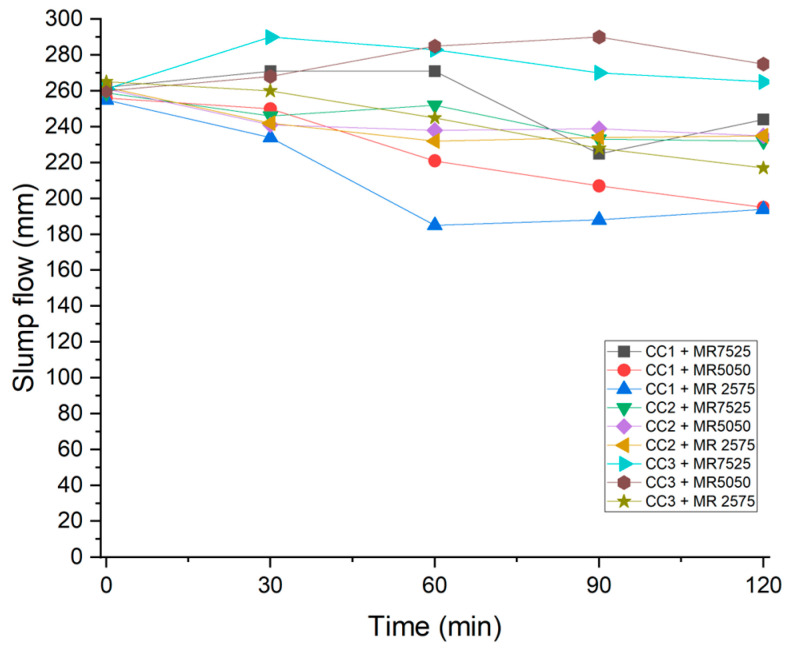
Slump loss behavior of calcined clays containing different superplasticizer samples.

In calcined clay cements, fluid retention is one of the main limitations that needs to be overcome, and the mechanisms involved in their rapid loss of fluidity remain unknown [1]. In order to evaluate the fluid retention of superplasticizers in calcined clay pastes suspended in SCPS, the spreading was measured every 30 min, for 2 h, as can be seen in Figure 14.

For CC1 clay, it can be observed that with the increase in the maintenance polymer content in the superplasticizer, the slump flow of the paste decreased more rapidly. In pastes with CC2 clay, the superplasticizers showed similar behavior, regardless of the dispersion and maintenance polymer contents. Finally, in pastes with CC3 clay, the superplasticizers MR7525 and MR5050 showed better performance in terms of slump retention, while MR2575, with a higher content of maintenance polymer, showed greater slump flow loss.

The mixtures that showed the greatest slump loss were those with additives MR7525 and MR5050 and calcined clay CC1, which has a higher amount of metakaolinite and a higher specific surface area (BET). Calcined clay CC3, which has an intermediate amount of metakaolinite and a lower specific surface area (BET), showed greater slump retention with additives MR7525 and MR5050. Clay CC2, which has a lower amount of metakaolinite and an intermediate specific surface area (BET), showed the least variation in slump retention.

The presence of clay minerals causes a decrease in the fluidity of the paste. This happens because high-valence metal ions, such as Al^3+^ and Si^4+^, which make up the clay structure, are easily replaced by lower-valence ions—giving the clay surface a negative charge. Consequently, some of the Ca^2+^ ions present in the paste, due to the SCPS, are attracted to this surface, rapidly reducing the fluidity of the mixture [95]. Some authors [21,96] have found that the greater the amount of the same clay minerals in the composition of composite cements, the greater the loss of slump in these samples.

**Figure 15:**
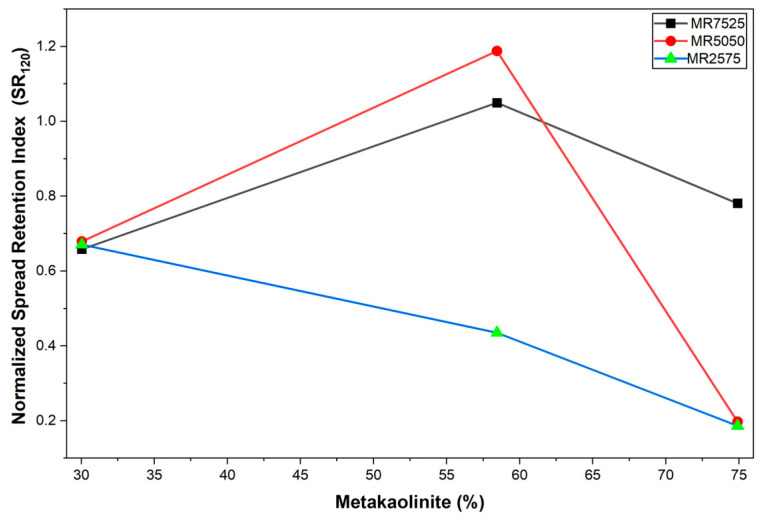
Correlation between the percentage of metakaolinite and the normalized spread retention index.

Figure 15 shows the normalized spread retention index (*SR*_120_) as a function of metakaolinite content for the three investigated PCEs. At a low metakaolinite content (30.04 wt%), all admixtures present similar *SR*_120_ values, indicating comparable dispersion efficiency. With increasing metakaolinite content, the performance of the PCEs diverges. MR7525 and MR5050 exhibit higher *SR*_120_ values at approximately 58.45 wt% metakaolinite, with MR5050 showing values slightly above unity, which can be attributed to delayed dispersion and progressive deflocculation effects. In contrast, MR2575 shows a continuous decrease in *SR*_120_, reflecting a higher sensitivity to clay–PCE interactions. At a high metakaolinite content (74.91 wt%), a pronounced reduction in spread retention can be observed for MR5050 and MR2575 admixtures, indicating a critical metakaolinite threshold beyond which PCE efficiency is significantly reduced. MR7525 maintains comparatively higher *SR*_120_ values under these conditions, suggesting a more robust molecular architecture against clay–polymer interactions.

Overall, the normalized spread retention index provides a quantitative framework linking clay reactivity and PCE molecular architecture. The superior performance of MR7525 at high metakaolinite contents directly reflects its longer effective main chain and lower side-chain density identified by ^1^H NMR, whereas the rapid loss of fluidity observed for MR2575 is consistent with its higher branching and functional group density, which enhances adsorption but limits steric stabilization.

### 3.5. Zeta Potential of Calcined Clays Containing Different Superplasticizer Samples

**Figure 16:**
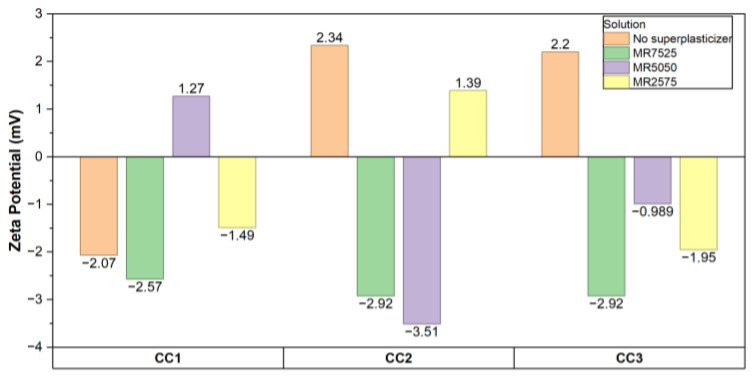
Zeta potentials of calcined clays in SCPs with and without superplasticizers.

Zeta potential analyses were performed on clay suspensions in synthetic cement pore solutions (SCPSs) under conditions with and without the addition of polycarboxylate ether (PCE)-based superplasticizers. For each calcined clay, the samples in the series “without superplasticizer”, “MR7525”, “MR5050”, and “MR2575” were measured. The numerical values obtained are shown in Figure 16.

Note that, in all samples, the zeta values showed very low magnitudes (0.0–3.5 mV), which indicates that the electrical layer is strongly compressed by the high ionic strength of the SCPS and/or by the concentrations of Ca^2+^ present, as reported for clays subjected to similar conditions [24,88]. Comparing the conditions with and without PCE additives, there was no consistent pattern of shifts to more negative or positive values. In CC2, the condition without PCE resulted in +2.34 mV, while with MR5050 a value of −3.51 mV was obtained. In CC1, the addition of MR5050 caused a change to +1.27 mV.

These results confirm that, under the high-ionic-strength conditions of SCPS, zeta potential is not a reliable descriptor of dispersion efficiency and that the differences in performance among the investigated PCEs are predominantly governed by steric effects associated with their molecular architecture. Although, under certain conditions, direct adsorption of PCE carboxylate groups can increase the negative magnitude of zeta potentials [97] and under others Ca^2+^-mediated ionic bridging or near-neutral surface charges may limit this effect [60,98], such electrostatic variations do not translate into the distinct dispersing behaviors observed in the present system.

## 4. Conclusions

The results obtained in this study demonstrate that the rheological performance of calcined clay suspensions in synthetic cement pore solutions (SCPSs) is governed by a complex combination of the physicochemical characteristics of the clays and the molecular structure of polycarboxylate ether (PCE)-based superplasticizers. The additive demand, fluidity maintenance, and electrokinetic behavior are profoundly influenced by the specific surface area, metakaolinite content (ranging from 30.04% in CC2 to 74.91% in CC1), Blaine fineness (7.438 to 13.116 m^2^·g^−1^), and particle size distribution of each sample, as well as by the ratio between dispersing and maintenance polymers in the PCEs (from 75:25 in MR7525 to 25:75 in MR2575).

The superplasticizer dosage required to achieve a spread of 260 ± 5 mm varied significantly among the samples, highlighting the strong influence of fineness and particle morphology. Clays with a higher Blaine fineness and a higher metakaolinite content, such as CC1 (74.91% metakaolinite, Blaine: 9.720 m^2^·g^−1^) and CC3 (58.45% metakaolinite, Blaine: 13.116 m^2^·g^−1^), required markedly higher PCE dosages (up to two times those for CC2) to reach target fluidity, reflecting their greater reactivity and high adsorption capacity. In contrast, CC2 (30.04% metakaolinite, Blaine: 7.438 m^2^·g^−1^) exhibited lower water demand (0.75 SCPS/clay ratio for 180 ± 5 mm spread without PCE) and lower PCE dosages overall.

In terms of slump maintenance, the loss of fluidity over 120 min was directly related to the mineralogy and surface area of the clays, as quantified by the normalized spread retention index (*SR*_120_). For a low metakaolinite content (CC2, 30.04 wt%), all PCEs maintained similar *SR*_120_ values close to 1, indicating minimal fluidity loss. At an intermediate metakaolinite content (58.45 wt%, CC3), MR7525 and MR5050 achieved *SR*_120_ > 1 (with MR5050 slightly higher due to delayed dispersion effects), while MR2575 showed reduced retention. At a high metakaolinite content (74.91 wt%, CC1), *SR*_120_ dropped significantly for MR5050 and MR2575 (rapid spread reduction), whereas MR7525 sustained comparatively higher values, demonstrating greater robustness against excessive adsorption in highly reactive systems. Although the higher maintenance polymer fraction in MR2575 was intended to delay fluidity loss, it proved less effective in high-metakaolinite clays, where initial adsorption dominated.

Zeta potential analyses showed values close to zero (−3.5 to +3.5 mV) in the high-ionic-strength SCPS, regardless of PCE addition, with no consistent shift toward more negative magnitudes. This confirms that electrostatic mechanisms play a secondary role and that dispersion is primarily driven by steric hindrance from PCE side chains and Ca^2+^-mediated adsorption.

Based on these findings, it is suggested that future studies include the quantification of the total organic carbon (TOC) content of clays, since residual organic matter can significantly influence the adsorption of PCEs and the additive demand. Additionally, it is recommended to carry out complete rheometric tests (stress–strain curves and apparent viscosity) to characterize more precisely the flocculation and deflocculation mechanisms in the interaction between clays and PCEs. Such analyses will allow for a deeper understanding of the simultaneous influence of clay characteristics and superplasticizer architecture on rheological behavior.

In summary, this study shows that PCE performance strongly depends on the compatibility between clay mineralogy (particularly metakaolinite content) and polymer architecture (dispersing vs. maintenance ratio). For clays with low metakaolinite contents (30.04%), any tested PCE provides adequate retention; for higher contents (58.45–74.91%), PCEs with higher dispersing polymer fractions (e.g., MR7525) offer superior fluidity retention and lower sensitivity to reactivity. These quantitative insights provide practical guidelines for selecting or designing optimized PCEs for cements containing high proportions of calcined clay, improving workability and reducing dosage requirements in sustainable low-clinker systems.

## Figures and Tables

**Table 1:** Density, solids content, and pH of PCE superplasticizers.

PCE Superplasticizer	Density (g/cm^3^)	Solids Content (wt%)	pH
MR7525	1.093	48.14	4.07
MR5050	1.090	48.38	4.40
MR2575	1.086	48.61	4.73

**Table 2:** FTIR of PCE superplasticizers.

Peak	Number Wave	Functional Group	References
(1)	3377	O–H bands of water; N–H stretching vibrations	[48]
(2)	2920	C–H stretching vibration	[20,43,44,49,50]
(3)	2883	C–H stretching vibration	[20,43,44,49,50]
(4)	1640	C=O bonds or –C=C– vibration adsorption	[20,44]
(5)	1456	CH_2_ stretching vibration	[43,49]
(6)	1349	CH_3_ stretching vibration	[49]
(7)	1288	C–H stretching vibration	[44]
(8)	1251	C–O stretching	[49]
(9)	1081	C–O–C stretching	[20,48,50,51]
(10)	948	–COO^−^ groups	[44]

**Table 3:** Chemical shifts and integral areas of the ^1^H NMR of HEPG additives.

Superplasticizer	Shifts (ppm)	Integral Areas	Integral Areas (%)
	4.752	5999.982	14.46
MR7525	3.654	14,517.088	34.98
	3.015	6636.359	15.99
	1.726	8829.141	21.27
	1.328	3039.371	7.32
	0.856	2479.944	5.98
	4.673	3523.633	9.40
MR5050	3.661	14,589.963	38.94
	2.769	6293.350	16.80
	1.751	7734.059	20.64
	1.424	3136.013	8.37
	0.861	2188.778	5.84
	4.729	5249.557	12.89
MR2575	3.646	14,518.176	35.64
	2.628	8246.229	20.25
	1.836	3676.913	9.03
	1.548	5364.164	13.17
	0.895	3676.011	9.03

**Table 4:** Molecular mass, PDI, and conversion rate of PCE superplasticizer samples.

PCE Superplasticizer	M_n_ (g·mol^−1^)	M_w_ (g·mol^−1^)	PDI (M_w_/M_n_)	Conversion Rate (%)
MR7525	10.205	12.749	1.249	79.00
MR5050	9.989	12.241	1.225	74.20
MR2575	14.812	19.892	1.343	88.37

**Table 5:** Chemical and physical characterization of raw clays and calcined clays.

Chemical Characteristics (wt%)	C1	C2	C3	CC1	CC2	CC3
SiO_2_	53.00	65.00	53.00	52.00	67.00	53.00
Al_2_O_3_	41.00	24.00	38.00	43.00	24.00	39.00
Fe_2_O_3_	1.80	4.90	4.50	1.60	4.30	4.00
TiO_2_	2.40	0.92	2.20	2.10	0.79	2.10
Na_2_O	0.06	0.74	0.00	0.06	0.87	0.04
MgO	0.16	0.74	0.40	0.11	0.76	0.46
K_2_O	0.73	1.70	1.40	0.65	1.60	1.40
CaO	0.13	0.91	0.03	0.08	0.69	0.05
Others	0.47	0.80	0.33	0.39	0.87	0.34
LOI	13.81	11.15	13.28	2.92	2.77	2.68
SiO_2_ + Al_2_O_3_ + Fe_2_O_3_	95.80	93.90	95.50	96.60	95.30	96.00
Al_2_O_3_/SiO_2_	0.77	0.37	0.72	0.83	0.36	0.74
Kaolinite content (by TGA)	77.03	30.36	60.08	2.12	0.32	1.63
Metakaolinite content (by TGA)	-	-	-	74.91	30.04	58.45
Physical Characteristics						
D_10_ (μm)	2.04	1.86	1.29	2.86	2.24	2.34
D_50_ (μm)	11.9	12.3	7.89	19.8	19.8	16.3
D_90_ (μm)	43.2	54.6	29.6	59.7	73.1	56.7
D_m_ (μm)	19.7	23.4	14.6	28.5	32.7	26.0
Blaine fineness (m^2^·g^−1^)	-	-	-	9.720	7.438	13.116
Density (g·cm^−3^)	2.60	2.50	2.65	2.60	2.57	2.60
BET specific area (m^2^·g^−1^)	27.8	48.0	31.7	48.4	33.7	28.4
Liquid limit (%)	53	47	56	44	-	43
Plasticity index (%)	17	21	26	10	-	11

**Table 6:** Minerals identified and their corresponding PDF numbers from the ICDD database.

Minerals		C1/CC1	C2/CC2	C3/CC3
1	Muscovite	99-100-5709	99-100-5709	99-100-6210
2	Kaolinite	99-101-0867	99-101-0867	99-101-0867
3	Quartz	99-100-4261	99-100-5716	99-101-1684
4	Anatase	99-100-9705	99-101-0679	99-100-9706
5	Orthoclase	-	99-100-0318	-
6	Rutile	99-100-1732	99-100-1732	99-100-1733
7	Gibbsite	99-100-9730	-	99-100-9730

## Data Availability

The original contributions presented in this study are included in the article. Further inquiries can be directed to the corresponding author.
